# Layperson Perception of Reflux‐Related Symptoms

**DOI:** 10.1002/oto2.51

**Published:** 2023-05-09

**Authors:** Jakob L. Fischer, Anthony M. Tolisano, Alvaro I. Navarro, Lily Trinh, Waleed M. Abuzeid, Ian M. Humphreys, Nadeem A. Akbar, Sharan Shah, John S. Schneider, Charles A. Riley, Edward D. McCoul

**Affiliations:** ^1^ Department of Otolaryngology–Head and Neck Surgery Walter Reed National Military Medical Center Bethesda Maryland USA; ^2^ Department of Surgery Uniformed Services University of the Health Sciences Bethesda Maryland USA; ^3^ Department of Otolaryngology–Head and Neck Surgery Tulane University New Orleans Louisiana USA; ^4^ Division of Rhinology and Endoscopic Skull Base Surgery, Department of Otolaryngology–Head and Neck Surgery University of Washington Seattle Washington USA; ^5^ Division of Rhinology and Skull Base Surgery, Department of Otorhinolaryngology–Head and Neck Surgery Albert Einstein College of Medicine Bronx New York USA; ^6^ Department of Otolaryngology–Head and Neck Surgery Washington University School of Medicine St. Louis Missouri USA; ^7^ Department of Otorhinolaryngology and Communication Sciences Ochsner Clinic Foundation New Orleans Louisiana USA

**Keywords:** definition of terms, gastro‐esophageal reflux disease, GERD, health literacy, patient‐provider communication, reflux, symptom, word‐association

## Abstract

**Objective:**

To assess for differences of intended meaning in the description of reflux‐related symptoms among otolaryngology patients and clinicians.

**Study Design:**

Cross‐sectional survey‐based study.

**Setting:**

Five tertiary, academic otolaryngology practices.

**Methods:**

Between June 2020 and July 2022, a questionnaire consisting of 20 common descriptors of reflux‐related symptoms within four domains (throat‐, chest‐, stomach‐, and sensory‐related symptoms) was completed by patients. Attending otolaryngologists at five academic medical centers then completed the same survey. The primary outcome was to assess differences in patient and clinician perceptions of reflux‐related symptoms. Differences based on geographic location was a secondary outcome.

**Results:**

A total of 324 patients and 27 otolaryngologists participated. Patients selected a median of six terms compared with 10.5 for otolaryngologists (*p* < .001). Otolaryngologists were more likely to select sensory symptoms (difference: 35.8%; 95% confidence interval [CI]: 19.2%, 52.4%), throat‐related symptoms (32.4%; 21.2, 43.6%), and chest‐related symptoms (12.4%; 8.8, 15.9). Otolaryngologists and patients were equally likely to consider stomach symptoms as related to reflux (4.0%, −3.7%, 11.7%). No significant differences were identified based on geographic location.

**Conclusion:**

There are differences between otolaryngologists and their patients in the interpretation of the symptoms of reflux. Patients tended to have a narrower interpretation of reflux with symptoms primarily limited to classic stomach‐related symptoms, while clinicians tended to have a broader definition of reflux that included extra‐esophageal manifestations of disease. This has important counseling implications for the clinician, as patients presenting with reflux symptoms may not comprehend the relationship of those symptoms to reflux disease.

Reflux‐related symptoms are common complaints among patients treated in both the primary and emergency care settings in the United States. Each year, over 9 million emergency department visits and 4.5 million primary care visits are related to gastro‐esophageal reflux disease (GERD),^1^ accounting for annual medical cost expenditures in excess of 10 billion dollars per year.^2^ Up to 40% of Western adult populations report chronic heartburn or regurgitation symptoms associated with reflux,^3^, ^4^ while GERD has been associated with significant loss of work and activity days.^4^ Despite its prevalence, the exact symptoms associated with this disease process remain incompletely characterized. This has significant implications for patient and clinician communication and may result in frustration, delays in care, and misdiagnosis.

In 2006, an international consensus group, the Montreal Classification, defined GERD as a condition that “develops when the reflux of stomach contents causes troublesome symptoms and/or complications.” These troublesome symptoms were subsequently divided into thirteen constituent categories that were either esophageal or extra‐esophageal in nature and ranged from the “typical reflux syndrome” to associated conditions such as recurrent otitis media and reflux cough syndrome.^5^ Despite this, diagnosis based on extraesophageal symptoms remains difficult and there is disagreement about the degree to which certain extraesophageal symptoms relate to reflux. The American College of Gastroenterology notes that while numerous extraesophageal symptoms have been associated with GERD, the ability to establish a causal link has been difficult.^6^ With these ongoing debates, defining the symptoms associated with GERD is difficult and underscores the potential for different definitions between patients and clinicians.

Health literacy describes the extent to which patients understand information provided by health care professionals. Patients with poorer health literacy demonstrate worse overall outcomes and higher mortality.^7^ Health literacy is, in part, related to receptive communication^8^ and differences in interpretations. Expectations regarding the management of reflux‐related symptoms may be variable as semantic misunderstandings may increase barriers to effective communication. Otolaryngology patients in general,^9^ and rhinologic patients specifically,^10^ appear to have high levels of health literacy. Other otolaryngologic complaints such as congestion,^11^ dizziness,^12^ and sinus infections^13^ have demonstrated significant variability and differences in interpretation of common symptoms between clinicians and patients.

The primary objective of this study was to evaluate the potential differences between patients and clinicians in defining the symptoms related to reflux. Secondary objectives included assessing differences in symptoms associated with reflux based on geographic location.

## Materials and Methods

### Study Population

Study participants in this cross‐sectional survey study were consecutive adult patients (>18 years) presenting for routine clinical care for any chief complaint at outpatient otolaryngology clinics who were willing to complete a brief questionnaire before their clinical encounter. Enrollment occurred between June 2020 and June 2022 at five academic medical centers. The Walter Reed National Military Medical Center Institutional Review Board approved the study protocol (WRNMMC‐EDO‐2019‐097). Demographic data was collected including age, gender, race/ethnicity, zip code, and level of education. Patient zip code was used to determine whether an individual resided in an urban or rural setting based on 2010 census classification of urban and rural areas.^14^ The same questionnaire was also administered to otolaryngology faculty who were categorized as clinicians.

### Questionnaire Development and Administration

A list of survey items was compiled from discussions with patients, medical colleagues, and literature review to capture a range of symptoms that may be used to characterize reflux. Items were then selected for inclusion following group discussion facilitated by the senior authors (C.A.R. and E.D.M.). The final survey contained 20 possible symptoms which were randomly arranged into a 5 × 4 grid on the center of a piece of paper to reduce lead‐item preference. Participants were asked to circle as many items as they required to answer the question “What are the symptoms of reflux?”

The 20 symptoms were then grouped into four broad categories for the purpose of analysis: throat symptoms (hoarseness, phlegm, sore throat, trouble swallowing, lump in throat, foreign body, and painful swallowing), chest symptoms (cough, thick mucus, and heartburn), stomach symptoms (feeling bloated, nausea, indigestion, gas, regurgitation, sour stomach, upset stomach, belly ache, and belching), and sensory symptoms (bad taste).

Patients were asked to complete the survey prior to encountering the clinicians in order to reduce the risk of influencing the results. Patients were instructed to select as many symptoms from the list as they felt were related to reflux, whether the individual had personally experienced any of the symptoms themselves. Clinicians were provided the survey to complete during scheduled departmental academic conferences.

### Statistical Analysis

The surveys were anonymous and contained no personally identifiable information. The number of patients and clinicians who selected each survey response as well as relationships among responses were analyzed. Patient and clinician responses were analyzed to identify patterns among symptom domains and demographic variables. Univariate analysis of group differences among categorical variables was performed. Confidence intervals were calculated to determine differences in responses between patient and clinician populations. All tests used a significance level of ≤.05. A heat map was generated by uploading anonymous matrix data files to Heatmapper^15^ which calculated a pairwise distance matrix using Euclidian measurements.

## Results

**Figure 1:**
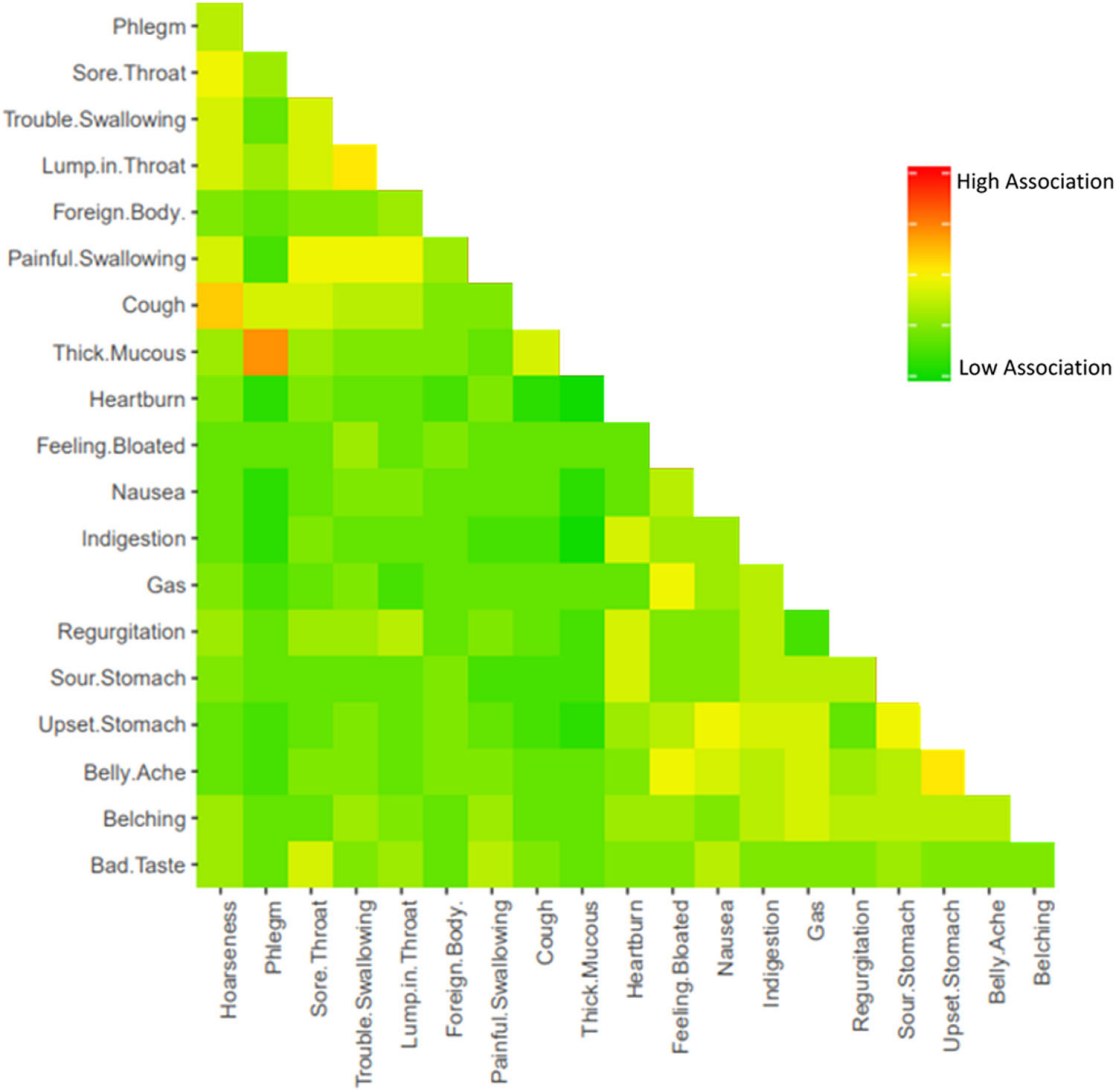
Heat map displaying association of individual survey items. Thick mucus and phlegm, and cough and hoarseness demonstrated the highest association

A total of 324 patients across five different geographic regions (Washington DC, New Orleans, St. Louis, Seattle, and New York) participated in the study, of which 183 (56.5%) were female with a mean (SD) age of 51.0 (16.6) (Table 1). Item responses were tabulated for each individual symptom as well as for the four broad categories. A symptom category was considered to be positive if one or more of the component definitions was circled by the respondent. Patients associated stomach symptoms most commonly with reflux (299, 92.3%), followed by chest symptoms (284, 87.7%), throat symptoms (195, 60.2%), and sensory symptoms (136, 32.0%) (Table 2). Greater than 50% of patients associated reflux with the three specific symptoms of heartburn (253, 78.1%), indigestion (192, 59.3%), and regurgitation (191, 59.0%). Most patients included symptoms from more than one symptom group (301, 92.9%). Only 23 patients (7.1%) included symptoms within only one category; of those 23 patients, 14 (60.9%) specified stomach‐related symptoms. Pairwise distance matrix mapping demonstrated few notable associations among individual symptoms. The strongest associations were between thick mucus and phlegm and cough and hoarseness. Other more commonly associated symptoms were lump in throat and trouble swallowing as well as upset stomach and belly ache (Figure 1).

**Table 1:** Patient and clinician demographics

				Patients by geographic location, n (%)					
	Clinicians, n (%)	All patients, n (%)	*p* Value	Washington, DC	New Orleans	Seattle	St. Louis	New York City	*p* Value
Total number	27	324		100	105	50	36	33	
Mean age	37.6 ± 11.1	51.0 ± 16.6	<.0001	47.6 ± 15.7	54.3 ± 14.4	48.9 ± 19.5	48.2 ± 17.4	55.7 ± 17.3	.0085
Gender									
Male	23 (85.2)	141 (43.5)	<.0001	54 (54)	46 (43.8)	26 (52)	15 (41.7)	8 (24.4)	
Female	4 (14.8)	183 (56.5)		46 (46)	59 (56.2)	24 (48)	21 (58.3)	25 (75.6)	
Race									
Caucasian	25 (92.6)	214 (66.1)	.0045	70 (70)	71 (67.6)	41 (82)	25 (69.4)	7 (21.1)	.0002
African American	0 (0)	74 (22.8)		21 (21)	24 (22.9)	2 (4)	9 (25)	18 (54.6)	
Asian	2 (7.4)	11 (3.4)		4 (4)	4 (3.8)	2 (4)	0 (0)	1 (3)	
American Indian	0 (0)	1 (0.3)		1 (1)	0 (0)	0 (0)	0 (0)	0 (0)	
Other	0 (0)	17 (5.3)		4 (4)	5 (4.8)	3 (6)	0 (0)	5 (15.2)	
Ethnicity									
Hispanic	2 (7.4)	35 (10.8)	.583	12 (12)	10 (9.5)	4 (8)	0 (0)	9 (27.3)	.0156
Non‐Hispanic	25 (92.6)	289 (89.2)		88 (88)	95 (90.5)	46 (92)	36 (100)	24 (72.7)	
Highest education									
Elementary School	0 (0)	12 (3.7)	<.0001	0 (0)	10 (9.5)	0 (0)	0 (0)	2 (6.1)	.0234
High school	0 (0)	52 (16.1)		16 (16)	11 (10.5)	10 (20)	5 (13.9)	10 (30.3)	
College	0 (0)	158 (48.8)		45 (45)	54 (51.4)	24 (48)	21 (58.3)	14 (42.4)	
Graduate school	27 (100)	96 (29.6)		39 (39)	29 (27.62)	13 (26)	8 (22.2)	7 (21.2)	
Urban vs rural									
Urban	27 (100)	303 (93.5)	.171	97 (97)	101 (96.2)	41 (82)	32 (88.9)	32 (97)	.0075
Rural	0 (0)	21 (6.5)		3 (3)	4 (3.8)	9 (18)	4 (11.1)	1 (3)	

**Table 2:** Comparison of patient and clinician definitions of the symptoms associated with reflux according to individual symptom and domain

				Patients by geographic location, n (%)				
Symptoms	Clinician, n (%) (n = 27)	All patients, n (%) (n = 324)	Clinician to all patients difference, % (95% CI)	Washington DC (n = 100)	New Orleans (n = 105)	Seattle (n = 50)	St. Louis (n = 36)	New York (n = 33)
Throat‐related symptoms								
Total	25 (92.6)	195 (60.2)	32.4 (21.2, 43.6)	66 (66)	64 (61)	26 (52)	21 (58.3)	18 (54.6)
Hoarseness	21 (77.8)	76 (23.5)	54.3 (38.0, 70.7)	25 (25)	25 (23.8)	10 (20)	8 (22.2)	8 (24.2)
Phlegm	13 (48.2)	72 (22.2)	25.9 (6.5, 55.3)	31 (31)	20 (19.1)	9 (18)	6 (16.67)	6 (18.2)
Sore throat	19 (70.4)	107 (33)	37.4 (19.4, 55.3)	40 (40)	36 (34.3)	11 (22)	10 (27.8)	10 (30.3)
Trouble swallowing	19 (70.4)	80 (23.7)	45.7 (27.8, 63.5)	26 (26)	23 (21.9)	12 (24)	9 (25)	10 (30.3)
Lump in throat	22 (81.5)	64 (19.8)	61.7 (46.5, 77.0)	22 (22)	22 (21)	7 (14)	4 (11.1)	9 (27.3)
Foreign body sensation	10 (37)	7 (2.2)	34.9 (16.6, 53.2)	3 (3)	4 (3.81)	0 (0)	0 (0)	0 (0)
Painful swallowing	10 (37)	48 (14.8)	22.2 (3.6, 40.8)	17 (17)	14 (13.3)	8 (16)	3 (8.3)	6 (18.2)
Chest‐related symptoms								
Total	27 (100)	284 (87.7)	12.4 (8.8, 15.9)	90 (90)	92 (87.6)	41 (82)	32 (88.9)	29 (87.9)
Cough	22 (81.5)	85 (26.2)	55.3 (40.0, 70.7)	24 (24)	28 (26.7)	12 (24)	10 (27.8)	11 (33.3)
Thick mucous	13 (48.2)	60 (18.5)	29.6 (10.3, 49.0	24 (24)	20 (19.1)	4 (8)	5 (13.9)	7 (21.2)
Heartburn	26 (96.3)	253 (78.1)	18.2 (9.8, 26.6)	82 (82)	81 (77.1)	36 (72)	29 (80.6)	25 (75.8)
Stomach‐related symptoms								
Total	26 (96.3)	299 (92.3)	4.0 (−3.7, 11.7)	91 (91)	100 (95.2)	42 (84)	34 (94.4)	32 (97)
Feeling bloated	8 (29.6)	85 (26.2)	*3.4* (*−14.5, 21.3)*	23 (23)	32 (20.5)	12 (24)	6 (16.7)	12 (36.4)
Nausea	5 (18.5)	100 (30.9)	*−12.4* (*−27.8, 3.15)*	21 (21)	38 (36.2)	14 (28)	12 (33.3)	15 (45.5)
Indigestion	22 (81.5)	192 (59.3)	22.2 (6.6, 37.8)	54 (54)	64 (61)	28 (56)	24 (66.7)	22 (66.7)
Gas	5 (18.5)	117 (36.1)	*−17.6* (*−2.1, −33.2)*	27 (27)	33 (31.4)	16 (32)	17 (32)	24 (72.7)
Regurgitation	21 (77.8)	191 (59)	18.8 (2.3, 35.4)	55 (55)	72 (68.6)	28 (56)	20 (55.6)	16 (48.5)
Sour stomach	13 (48.2)	138 (42.6)	*5.6* (*−14.1, 5.6)*	44 (44)	43 (41)	18 (36)	18 (50)	15 (45.5)
Upset stomach	6 (22.2)	134 (31.4)	*−19.1* (*−35.7, −2.6)*	33 (33)	40 (38.1)	21 (42)	20 (55.6)	20 (60.6)
Belly ache	2 (7.4)	66 (20.4)	*− 13.0* (*−23.8, −2.2)*	18 (18)	22 (21)	8 (16)	9 (25)	9 (27.3)
Belching	16 (59.3)	153 (47.2)	12.0 (−7.3, 31.4)	41 (41)	54 (51.4)	19 (38)	20 (46.1)	19 (57.6)
Sensory‐related symptoms								
Bad taste	21 (77.8)	136 (42)	35.8 (19.2, 52.4)	42 (42)	45 (42.9)	18 (36)	16 (44.44)	15 (45.5)

*Note*: Italics were our means of denoting significant (non‐italics) from non‐significant (italics) differences in patient populations.

Abbreviation: CI, confidence interval.

**Figure 2:**
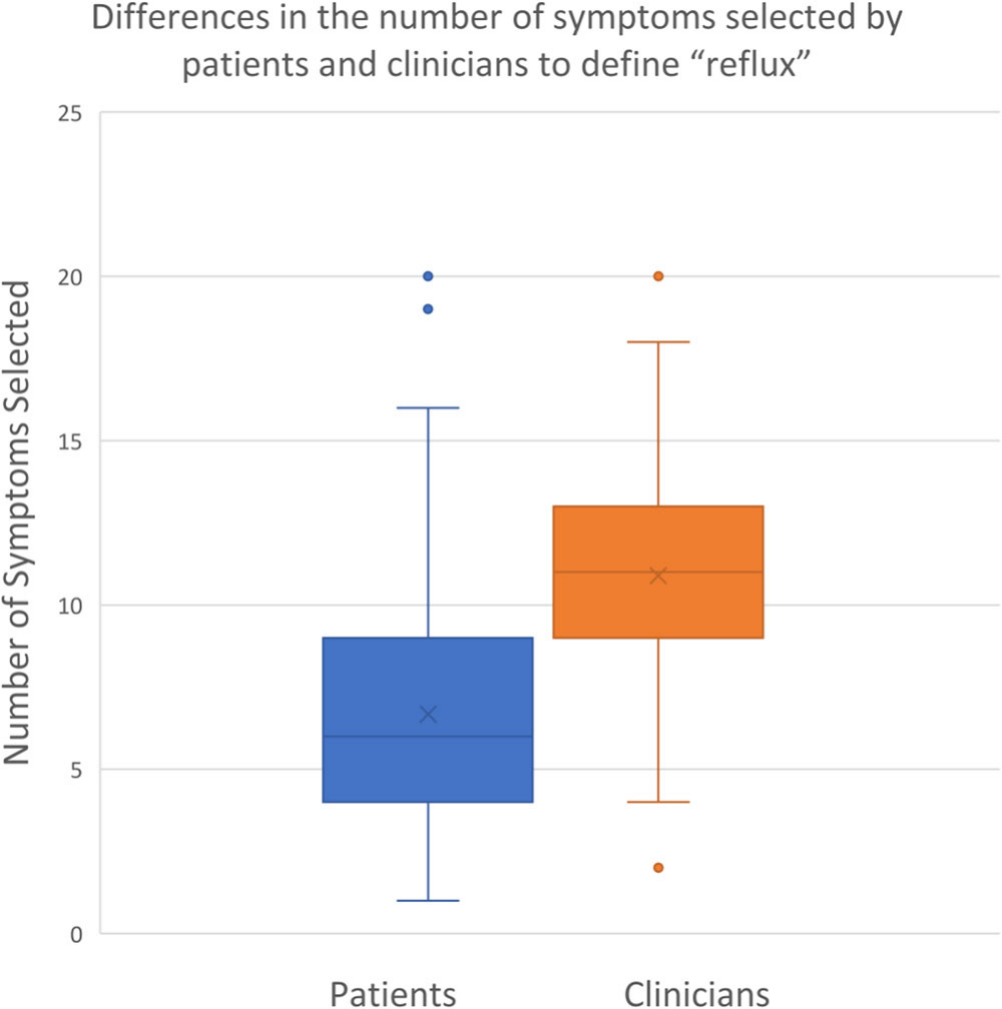
Differences in the number of symptoms selected by patients and clinicians to define “reflux.”

**Figure 3:**
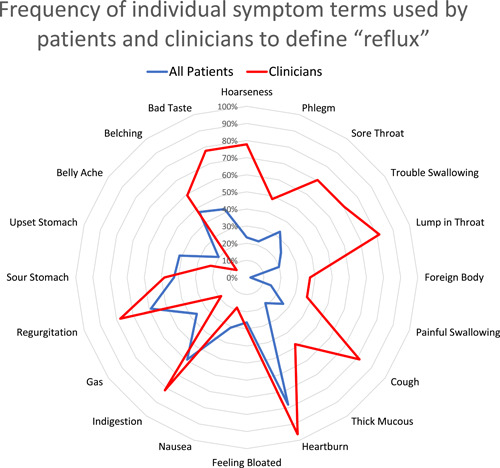
Frequency of individual symptom terms used by patients and clinicians to define “reflux.”

In total, 27 otolaryngologists completed the questionnaire. All otolaryngologists were attending clinicians. Otolaryngologists described the symptoms of reflux using a median of 11 (interquartile range [IQR]: 9‐13) symptoms when compared with 6 (IQR: 4‐9) symptoms in patients (*p* < .0001) (Figure 2). Otolaryngologists were more likely to describe reflux in terms of sensory symptoms (difference: 35.8%; 95% CI: 19.2%, 52.4%), throat‐related symptoms (difference: 32.4%; 95% CI: 21.2%, 43.6%), and chest‐related symptoms (difference 12.4%; 95% CI: 8.8%, 15.9%) (Table 2). The difference between otolaryngologists and patients was not significant with respect to stomach‐related symptoms (difference: 13.2%; 95% CI: −3.4%, 29.9%). Some specific stomach‐related symptoms were more often used by patients than clinicians, whereas in the other symptom categories all terms were used more frequently by clinicians (Figure 3).

**Figure 4:**
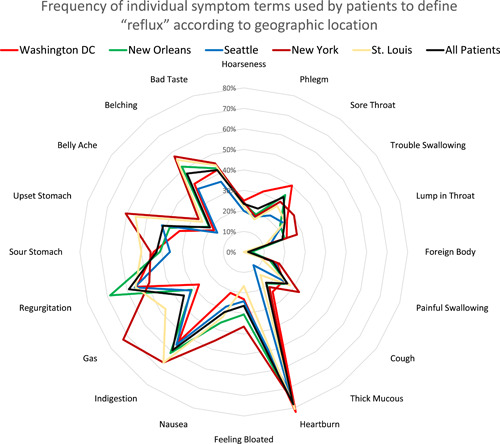
Frequency of individual symptom terms used by patients to define “reflux” according to geographic location

Geographic location did not have a significant impact on patient perception of symptoms of reflux. When comparing clinicians to patients of different geographic areas, clinicians remained more likely to describe reflux using throat‐related, chest‐related, and sensory‐related symptoms compared with patients (Table 3). No significant difference was observed between patients and clinicians in describing stomach‐related symptoms. When examining the patient populations only on the basis of individual symptoms, patients in New York appeared more likely to describe reflux using the symptom of gas, without any appreciable other differences noted (Figure 4).

**Table 3:** Percent differences between patients and clinicians and geographic regions and clinicians by symptom domain

	Clinicians vs patients by geographic location, % difference (95% CI)				
Symptom domain	Washington DC	New Orleans	Seattle	St. Louis	New York City
Throat symptoms	*26.6* (*13.0, 40.2)*	*31.6* (*18.1, 45.2)*	*40.6* (*23.6, 57.6)*	*34.3* (*15.4, 53.2)*	*38.1* (*18.4, 57.7)*
Chest symptoms	*10.0* (*4.1, 15.9)*	*12.4* (*6.1, 18.7)*	*18.0* (*7.4, 28.7)*	*11.1* (*0.8, 21.4)*	*12.1* (*1.0, 23.3)*
Stomach symptoms	5.3 (−3.8, 14.4)	1.1 (−7.2, 9.3)	12.3 (−0.1, 24.7)	1.9 (−8.5, 12.2)	−0.67 (−9.9, 8.5)
Sensory symptoms	*35.8* (*17.4, 54.2)*	*34.9* (*16.6, 53.2)*	*31.8* (*21.2, 62.3)*	*33.3* (*10.8, 55.9)*	*32.3* (*9.2, 55.4)*

*Note*: Italics were our means of denoting significant (non‐italics) from non‐significant (italics) differences in patient populations.

Abbreviation: CI, confidence interval.

## Discussion

The diagnosis of GERD is dependent on the subjective reporting of reflux‐related symptoms, despite the likelihood that patients and clinicians have differing opinions on what symptoms can be related to reflux. This disparity is present for several reasons but represents an area where communication can be limited^16^ and patient and clinician satisfaction can be negatively impacted.^17^ This study sought to evaluate the differences between patient and clinician interpretations of what constitutes the symptoms associated with “reflux” in an effort to identify potential barriers to communication.

The results support our hypothesis that patients and clinicians describe reflux differently. Moreover, there were not substantial differences in patient definitions based on geographic location. Patients demonstrated a narrower definition of reflux when compared with clinicians and often limited the description to stomach‐related symptoms while clinicians were more willing to consider throat, chest, and sensory symptoms as reflux‐related.

Our data suggest that otolaryngologists are more likely to consider extra‐esophageal manifestations of reflux. There are several reasons this may be the case. Current guidelines for GERD management recommend empiric therapy for patients with typical symptoms of heartburn and regurgitation. Heartburn and regurgitation are reportedly the most reliable symptoms for making a presumptive diagnosis of erosive esophagitis with sensitivity and specificity ranging from 30% to 76% and 62% to 96% specificity.^18^ In these patients, empiric therapy was successful in up to 71.1% of patients reporting classic reflux symptoms.^19^ In our patient population, heartburn and regurgitation were two of the three most commonly noted symptoms used to describe symptoms associated with reflux and indicates a known association between these particular symptoms and the diagnosis of GERD. Patients with these symptoms of a very common disease entity are likely to present to a provider with typical symptoms of reflux and receive a treatment that is overall efficacious in reducing their symptoms.

This can be directly contrasted to those symptoms considered as extra‐esophageal manifestations of reflux. The Montreal consensus on GERD noted that extra‐esophageal symptoms are often non‐specific, multifactorial in nature, and can show a low predictive value for GERD in the absence of more typical symptoms such as reflux or heartburn or sustained response to PPIs.^5^ More recent data has estimated that up to one third of GERD patients may present with extra‐esophageal symptoms, the most common of which are non‐cardiac chest pain (23.1%), hoarseness (14.8%), bronchitis (14.0%), asthma (9.3%), and globus sensation (7.0%).^20^ In patients with extra‐esophageal symptoms, heartburn or regurgitation can be absent in up to 40% to 60% of asthmatics, 57% to 94% of those with otolaryngologic complaints, and 43% to 75% of those with chronic cough.^21^ Patients with isolated atypical reflux symptoms have demonstrated poorer efficacy with anti‐reflux medications than those with traditional symptoms.^22^ This has resulted in current guidelines recommending against empiric therapy without direct evidence of reflux on diagnostic testing or ruling out alternative diagnoses.^6^ Compared with typical reflux symptoms, extra‐esophageal symptoms are less common and respond poorer to standard medication regimens, which may contribute to differences between patients and clinicians in associating these symptoms as GERD‐related. Another consideration is that our clinician population is composed solely of otolaryngologists who much more commonly treat patients on the basis of extra‐esophageal symptoms and thus may have a different perspective on the symptoms of reflux than clinicians within other fields of medicine.

It is important to consider the availability of over‐the‐counter medication and the large amount of information available to the public through direct‐to‐consumer advertising (DCTA) and word of mouth and their potential impact on patient understanding of reflux. Currently, heartburn‐related over‐the‐counter pharmaceuticals are third in annual sales in the United States behind only analgesics and upper respiratory medications (including allergy).^23^ The majority of these medication packaging are self‐described to treat “heartburn” and “acid indigestion,” which were amongst the top three most cited symptoms by patients to be related to reflux.

In the United States, DCTA costs drug companies an estimated 6 billion dollars annually.^24^ It is uncertain how much of this spending is directly related to reflux‐medication products, but this advertising has demonstrable impacts on self‐treatment and the patient‐physician relationship. One study estimates that up to 10% of survey respondents who had viewed DCTA for GERD initiated a conversation that often resulted in a change of therapy.^25^ This form of advertising also has direct impacts on health literacy. One study evaluated the impact of DCTA advertising on public knowledge of GERD, during which a mock advertisement about GERD and proton pump inhibitors was shown to participants in public venues. These patients demonstrated a 15% increase in correct answers regarding the definition of GERD, 25% increase in knowledge of available medications for GERD, and 45% increase in knowledge of side effects of proton pump inhibitors.^26^

The current analysis found significant differences between clinician and patients on many definitions of what constitutes “reflux” and emphasizes the importance of careful history taking and patient education in clinic. Most patients and clinicians tended to agree that stomach‐related symptoms were highly associated with reflux, but patients were much less likely to attribute extraesophageal symptoms, or symptoms within the chest, throat, and sensory domains, as related to reflux. This is important to consider when discussing with patients, as additional counseling may be necessary to educate that reflux may be a contributing factor in the manifestation of these complaints.

In our patient population, there was remarkably little difference in patient responses across different geographic regions. This contrasts with previous studies that demonstrated that geographic and regional differences may be associated with differences in linguistic meaning and/or semantics.^27^, ^28^ This may be in part secondary to smaller sample sizes from some geographic areas and warrants additional study.

There are inherent limitations from this study design. Patient‐responded questionnaires are subject to misinterpretation, miscomprehension, response bias, limited participation, and hidden agenda associated with completion of paperwork. This is additionally a questionnaire that has not been subjected to validation testing. Five medical treatment facilities representing a geographically diverse population were included, but some geographic regions had comparatively few responses which may limit the ability to detect statistically meaningful differences in patient populations. This study additionally only explored the symptoms associated with the word “reflux” in patients presenting to an otolaryngology clinic. Further study would be required to explore the interpretation of these symptoms with primary care physicians and gastroenterology, where the symptoms associated with reflux may vary significantly. Moreover, in the present study, it is unknown which patients had experienced or presented with reflux‐related symptoms, which may aid in further refinement of the symptoms associated with reflux.

## Conclusion

There are differences in the interpretation of the symptoms of reflux between otolaryngologists and their patients. Patients tended to have a narrower interpretation of reflux with symptoms primarily consisting of classic stomach‐related symptoms of reflux, while clinicians tended to have a broader definition of reflux that included extra‐esophageal manifestations of disease. This has important counseling implications for the clinician, as patients presenting with reflux symptoms may not comprehend the relationship of those symptoms to reflux disease.

## Author Contributions

**Jakob L. Fischer**: acquisition, analysis, and interpretation of data, drafting and revision of manuscript to include drafting for critically important intellectual content, final approval of version to be published and accountability for all aspects of work; **Anthony M. Tolisano**: acquisition of data, drafting and revision of manuscript to include drafting for critically important intellectual content, final approval of version to be published and accountability for all aspects of work; **Alvaro I. Navarro**: acquisition of data, drafting and revision of manuscript to include drafting for critically important intellectual content, final approval of version to be published and accountability for all aspects of work; **Lily Trinh**: acquisition of data, drafting and revision of manuscript to include drafting for critically important intellectual content, final approval of version to be published and accountability for all aspects of work; **Waleed M. Abuzeid**: acquisition of data, drafting and revision of manuscript to include drafting for critically important intellectual content, final approval of version to be published and accountability for all aspects of work; **Ian M. Humphreys**: acquisition of data, drafting and revision of manuscript to include drafting for critically important intellectual content, final approval of version to be published and accountability for all aspects of work; **Nadeem A. Akbar**: acquisition of data, drafting and revision of manuscript to include drafting for critically important intellectual content, final approval of version to be published and accountability for all aspects of work; **Sharan Shah**: acquisition of data, drafting and revision of manuscript to include drafting for critically important intellectual content, final approval of version to be published and accountability for all aspects of work; **John S. Schneider**: acquisition of data, drafting and revision of manuscript to include drafting for critically important intellectual content, final approval of version to be published and accountability for all aspects of work; **Charles A. Riley**: conception and design, acquisition, analysis and interpretation of data, drafting and revision of manuscript to include drafting for critically important intellectual content, final approval of version to be published and accountability for all aspects of work; **Edward D. McCoul**: conception and design, acquisition, analysis and interpretation of data, drafting and revision of manuscript to include drafting for critically important intellectual content, final approval of version to be published and accountability for all aspects of work.

## Disclosures

### Competing interests

None.

### Funding source

None.
